# Waterproof Graphene-PVDF Wearable Strain Sensors for Movement Detection in Smart Gloves

**DOI:** 10.3390/s21165277

**Published:** 2021-08-04

**Authors:** Hossein Cheraghi Bidsorkhi, Alessandro Giuseppe D’Aloia, Alessio Tamburrano, Giovanni De Bellis, Maria Sabrina Sarto

**Affiliations:** 1 Department of Astronautical, Electrical and Energy Engineering, Sapienza University of Rome, Via Eudossiana 18, 00184 Rome, Italy; alessandrogiuseppe.daloia@uniroma1.it (A.G.D.); alessio.tamburrano@uniroma1.it (A.T.); giovanni.debellis@uniroma1.it (G.D.B.); mariasabrina.sarto@uniroma1.it (M.S.S.); 2 Research Center on Nanotechnology Applied to Engineering of Sapienza (CNIS), Sapienza University of Rome, Via Eudossiana 18, 00184 Rome, Italy

**Keywords:** graphene, PVDF, piezoresistive nanocomposites, strain sensor, smart glove, movement detection

## Abstract

In this work, new highly sensitive graphene-based flexible strain sensors are produced. In particular, polyvinylidene fluoride (PVDF) nanocomposite films filled with different amounts of graphene nanoplatelets (GNPs) are produced and their application as wearable sensors for strain and movement detection is assessed. The produced nanocomposite films are morphologically characterized and their waterproofness, electrical and mechanical properties are measured. Furthermore, their electromechanical features are investigated, under both stationary and dynamic conditions. In particular, the strain sensors show a consistent and reproducible response to the applied deformation and a Gauge factor around 30 is measured for the 1% wt loaded PVDF/GNP nanocomposite film when a deformation of 1.5% is applied. The produced specimens are then integrated in commercial gloves, in order to realize sensorized gloves able to detect even small proximal interphalangeal joint movements of the index finger.

## 1. Introduction

Nowadays, sensors are becoming increasingly important and their market is growing exponentially [1]. In fact, sensors are central to industrial and civil applications, being used for process and structural health monitoring and wearable devices [2]. Moreover, they play a key role in medical practice for diagnostics, critical care, human movement detection, and human-robot interaction [3,4]. In particular, one of the major challenges is the development of cost effective, lightweight, and comfortable smart gloves, i.e., high-tech wearable devices able to detect various hand gestures, including fingers bending [5]. Therefore, the demand for novel, high performance, and multifunctional flexible sensors is increasing enormously [6]. However, traditional strain sensors suffer from poor stretchability and sensitivity. In addition, complex preparation processes involved and high fabrication costs restrict their application and development [7]. Then, sensors are often prone to different weather conditions and moisture, compromising their response and accuracy [6]. Hence, many efforts are devoted to overcome these bottlenecks. Recently, strain sensors-based on the use of electrically conductive polymer composites have attracted great interest due to their light weight, flexibility, easy processing, and cost effectiveness [8]. In particular, the development of new carbon-based conductive nanofillers is fueling the research of graphene-based polymer composites showing a favorable combination of electrical properties with typical features of polymeric materials, such as lightness and easy workability [9,10,11]. Among the most interesting properties of these materials, the electro-mechanical ones stand out, as they allow the development of innovative sensors, overcoming the limitations of traditional materials [12]. Therefore, piezoresistive polymer composites are being strongly investigated for sensor applications [13].

Electronic transport in a polymer nanocomposite is mainly driven by the formation of a continuous nanofiller conductive path within the insulating matrix [14,15,16]. Nevertheless, high filler loadings generally result in low mechanical resistance and poor workability. In this framework, carbon-based nanofillers gained sustaining interest from the scientific community and, among them, two-dimensional graphene nanoplatelets (GNPs) are attracting ever growing interest since they are cost effective and easily processable with respect to other graphene-based nanofillers, such as carbon nanotubes (CNTs) or graphene oxides [17,18]. Briefly, GNPs can be regarded as tiny stacks of graphene layers, with thicknesses in the 1–10 nm range and lateral linear dimensions varying from about 1 µm up to 20–25 µm [17]. Due to their outstanding properties, GNP-filled polymer composites have been widely investigated in various engineering applications, including electromagnetic compatibility [18,19,20], structural sensing and monitoring [21,22,23,24]. Different polymer matrices have been explored, such as polystyrene [17,20], epoxy-based vinyl ester resins [18,19,24], polydimethylsiloxane [5,22], polyurethane [25], and polyvinylidene fluoride (PVDF) [23,24,26,27,28]. Among them, PVDF is garnering ever growing interest for sensing applications owing to its uncommon chemical resistance and thermal mechanical physical properties, including piezoresistive and piezoelectric ones [29,30]. Moreover, the hydrophobic features of PVDF are crucial for the realization of self-cleaning anti-biofouling waterproof sensors [26]. Hence, several studies reported on the improvement of electric, mechanical and electromechanical properties of PVDF composites by the combination of carbonaceous nanofillers [31,32,33,34].

Despite that, the electromechanical properties of PVDF composites loaded with different GNP loading concentrations have not been deeply studied. In fact, to the best of the authors’ knowledge, only a few publications deal with such properties of PVDF nanocomposites, most of them using different graphene-based nanostructures as fillers, such as carbon fibers or nanotubes [13,26,27,34]. For instance, P. Costa et al. investigated the piezoresistive behavior of PVDF-based composites filled with graphene oxide and single wall CNTs [13,34]. More recently, the influence of polymer functionalization of hydrogen exfoliated graphene has been assessed with the aim of providing a stable dispersion in the PVDF polymer matrix [26], while the possibility to tune the piezoresistive properties of PVDF/CNT composite through the addition of poly methyl methacrylate has been demonstrated in [27].

Nevertheless, the development of PVDF/GNP nanocomposite films suitable for the realization of highly sensitive strain sensors to be embedded in smart gloves is still an open issue. In fact, according to [11], it is not easy to reach suitable electrical conductivity values for sensing applications through the addition of GNP quantities lower than 5% wt with respect to the PVDF amount, compromising the mechanical properties of the resulting nanocomposite [13].

At present, different technologies are available for the production of sensor-based smart gloves. For instance, inorganic functional materials have been designed to be integrated in smart gloves [35], but they could not be stretched due to their brittleness and rigidity, and they require complex fabrication processes [5]. Recently, ink-jet printing has attracted great attention owing to its simple process but conductive inks are generally prone to clogging the nozzle [36]. Comparatively, screen printing techniques have shown many advantages since they allow direct patterning and mass production. However, conventional elastomeric films generally used as substrates have a low surface energy, resulting in poor adhesion between the substrate and the sensing ink [37]. As a consequence, it is still challenging to directly print onto an elastomer by screen printing for sensing purposes, especially when high stretchability and sensing ranges are required [4]. On the other hand, sensors made of ionic liquids are typically characterized by high stretchability but less sensitivity [37,38].

Within this context, conductive composites are garnering a lot of interest for sensor-based smart gloves since they are more compatible to human body movement detection [5]. Among them, carbon-based nanocomposites usually have a high initial resistance, compromising the sensitivity [37]. Hence, metal-based nanocomposites have been investigated [4,37] but they usually require expensive metallic nanoparticles, classified as critical raw materials by the European Commission [4].

The scope of this paper is to overcome some of the most common limitations, such as highly initial resistivity, less sensitivity, and the use of expensive critical raw materials or expensive nanofillers. Therefore, PVDF/GNP nanocomposite films with outstanding piezoresistive properties are produced and their application as strain sensors to be embedded in sensor-based smart gloves is investigated. Thanks to the fabrication route previously developed in [23] and further improved, it is demonstrated that it is possible to realize PVDF/GNP nanocomposite films suitable for strain detection with a total amount of GNP lower than 2% wt with respect to the PVDF total amount. This result is feasible owing to a perfect integration of GNPs inside the polymer matrix along with an optimal dispersion of the fillers, without agglomerations or defects. The produced nanocomposite films are characterized in terms of waterproofness, electrical, mechanical, and electromechanical properties, and their morphology is assessed through scanning electron microscopy (SEM).

The results show that the produced nanocomposite films are hydrophobic and their response to an applied deformation is consistent and reproducible. In particular, a relative resistance change greater than 40% at 1.5% of the applied strain is measured for the 1% wt loaded PVDF/GNP nanocomposite film. As a comparison, the relative resistance change recently measured at 0.35% of the applied strain in case of polymer functionalized graphene-based PVDF flexible sensor is around 3.5% [26]. Therefore, the produced PVDF/GNP nanocomposite films are suitable for the realization of waterproof and flexible sensors. As a demonstration, the nanocomposite films are integrated in commercial hand gloves, thus realizing smart gloves able to detect even small proximal interphalangeal joint movements of the index finger, as little as 5°. Other carbon-based flexible nanocomposite films recently integrated in sensor-based smart gloves have not been tested for bending angles lower than 30° [5] and most of them are characterized only for fully finger bend movements [25,36,37,38].

## 2. Materials and Methods

### 2.1. Production of PVDF/GNP Composite Films

GNPs are synthesized through thermal expansion of an acid base-modified graphite intercalation compound (GIC). In particular, a small amount of GIC undergo a thermal shock at around 1150 °C for a few seconds, resulting in a 200-fold volume increase. The obtained expanded graphite is tip-sonicated in polymer solvent for 20 min through an ultrasonic probe set in pulse mode (1 s on and 1 s off), leading to a homogenous suspension of GNPs [18].

Then, pure PVDF is dissolved in N,N-dimethylformamide (DMF) solvent for 2 h under magnetic agitation at 65 °C [39]. Successively, the previously synthesized GNPs are loaded to the PVDF solution and mixed for 1 h at the same temperature. The resulting polymer-GNP liquid mixture is casted onto a glass mold with the aim of producing nanocomposite films with a thickness between 20 and 40 μm, in order to obtain flexible films without compromising their mechanical properties. The glass mold filled with the polymer-GNP mixture is placed in an oven for 8 h at 80 °C in order to allow the solvent removal.

The produced nanocomposite films employ different weight concentrations of GNPs compared to the total PVDF amount, namely 0.5%, 0.75%, 1%, 1.5%, and 2%, as reported in Table 1. Moreover, the samples are named using the letters P and G to indicate PVDF and GNPs, respectively, and the numbers following the letters correspond to the GNP weight contents in percent with respect to the polymer amount.

**Figure 1:**
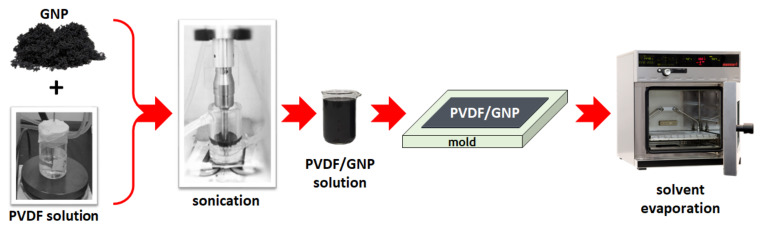
Sketch representing the main steps of the graphene-PVDF nanocomposite films production process.

Finally, the main steps of the nanocomposite films production process is summarized in Figure 1.

### 2.2. Morphological Characterization

The morphology of the produced nanocomposite specimens is investigated through a Zeiss Auriga Field Emission SEM. Fracture surfaces of the films are realized upon immersion in liquid nitrogen and, when required, the fractured films are sputter coated with a conductive 10 nm Cr layer by employing a Quorum Tech Q150T sputter coater.

### 2.3. Water Resistance Test

The hydrophobicity is assessed through the measurement of water contact angles (CAs). Such measurements are carried out using an optical CA meter and casting ∼2 μL water droplets on the coating surface. Then, the acquired pictures are processed using the ImageJ software version 1.53i (Image Processing and Analysis in Java).

### 2.4. Mechanical Characterization

**Figure 2:**
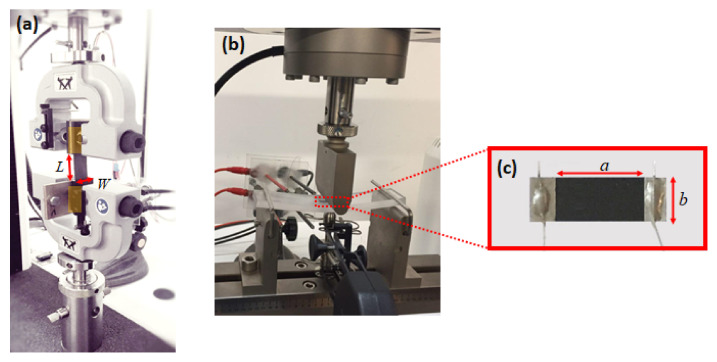
(**a**) Mechanical measurement test setup. Each rectangular strip has dimensions: L (gage length) = 5 cm and W = 2 cm. (**b**) Three-point bending test setup; (**c**) PVDF/GNP nanocomposite sample cut into a rectangular shape (*a* = 10 mm, *b* = 5 mm) with contact areas at both extremities.

The mechanical properties of the samples listed in Table 1 are assessed through mechanical tensile tests, carried out using an INSTRON 3366 universal testing machine. The samples to be tested are cut into rectangular strips, following a modified version of the standard ASTM D 882 [23]. The top and bottom parts of the specimens are inserted between the grips of a tensile test fixture, as shown in Figure 2a.

The Young’s modulus is extrapolated by dividing the longitudinal stress with the strain.

### 2.5. Electromechanical Characterization

The piezoresistive behavior of the samples are assessed by performing the three-point bending test, following the procedure described by the standard ASTM D790-03 and using the experimental setup sketched in Figure 2b.

In particular, the quasi static and cyclic mechanical load conditions are applied using an INSTRON 3366 universal testing machine. At the same time, a Keithley 6221 dc/ac current source and a Keithley 2182a nano-voltmeter are used to measure the electrical resistance *R* of the samples. With this aim, each PVDF/GNP nanocomposite sample is cut into a rectangular shape and two electrical contacts of areas of 4 × 2.5 mm are realized at both extremities, using commercial silver-paint and silver-based epoxy adhesive, following the procedure described in [23,24]. The resulting specimen, shown in Figure 2c, is then pasted over a polycarbonate beam.

Then, the relative resistance change Δ*R*/*R*_0_ is computed as the difference between the electrical resistance *R* and the initial resistance of the film *R*_0_ measured without any applied deformation, divided by *R*_0_. Then, the Gauge factor (GF) is given by the ratio of Δ*R*/*R*_0_ to the mechanical strain *ε* [24]:

(1) $$GF=\frac{\Delta R/R_{0}}{\varepsilon }$$

Finally, the effective dc electrical conductivity *γ_eff_* is computed as:

(2) $$\gamma_{eff}=a{\left(btR_{0}\right)}^{-1}$$

where *t* is the thickness reported in Table 1, *a* and *b* are the geometrical dimensions of the specimen (Figure 2c).

### 2.6. Sensorized Glove

**Figure 3:**
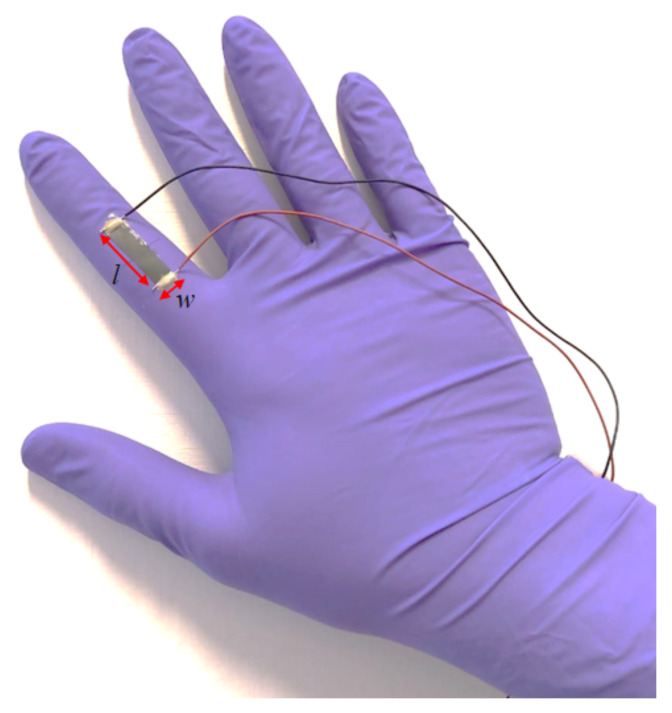
Picture of a nanocomposite film pasted on a commercial hand glove in order to detect index finger bending. The dimensions are *l* = 2.5 cm and *w* = 0.5 cm.

The fabricated nanocomposite films named PG-1 and PG-2 in Table 1 are cut and pasted on commercial hand gloves using common highly adhesive glue, with the aim of realizing two sensorized gloves able to detect the index finger bending. In particular, the selected rectangular shaped samples are pasted on the surfaces of gloves overlying the proximal interphalangeal joint of the index finger, as shown in Figure 3.

Successively, two thin silver-paint layers (Electrolube^®^) are deposited on rectangular areas of 5 × 2.5 mm at the extremities of the PVDF/GNP nanocomposite films. Then, the gloves are cured at room environment conditions for 2 days.

The electrical resistance *R* of the pasted nanocomposite film is measured following the procedure described in Section 2.5.

## 3. Results

### 3.1. Film Morphology

**Figure 4:**
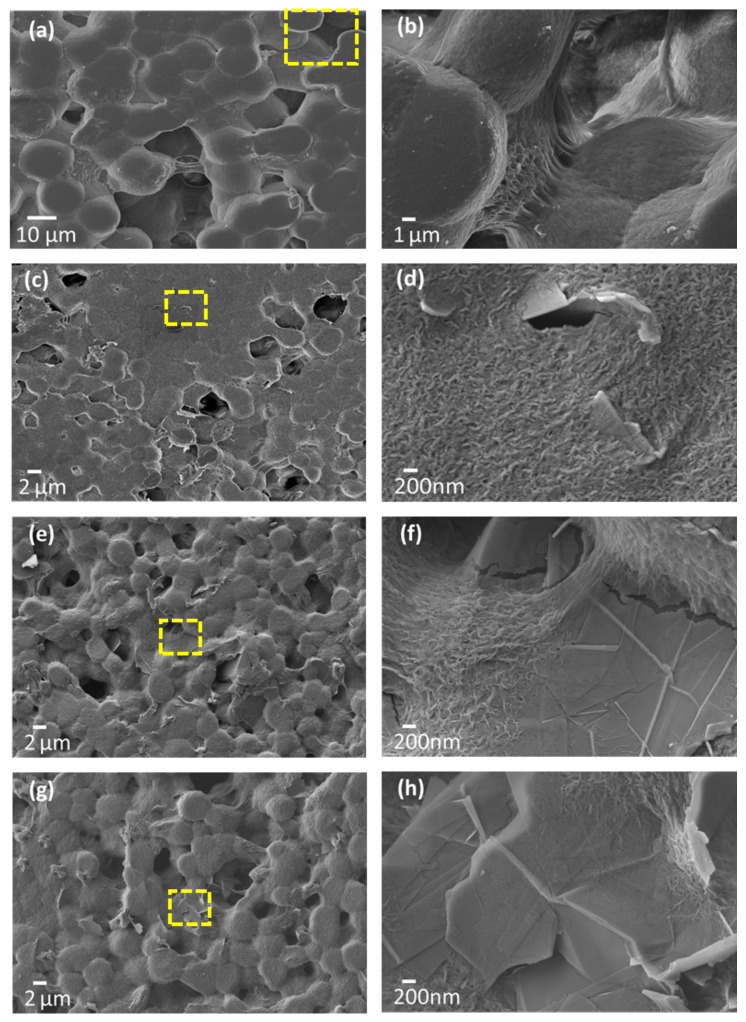
SEM micrographs of the top surface of neat PVDF (**a**,**b**) and PVDF/GNP composite films loaded at 1 (**c**,**d**), 1.5 (**e**,**f**), and 2% wt (**g**,**h**).

**Figure 5:**
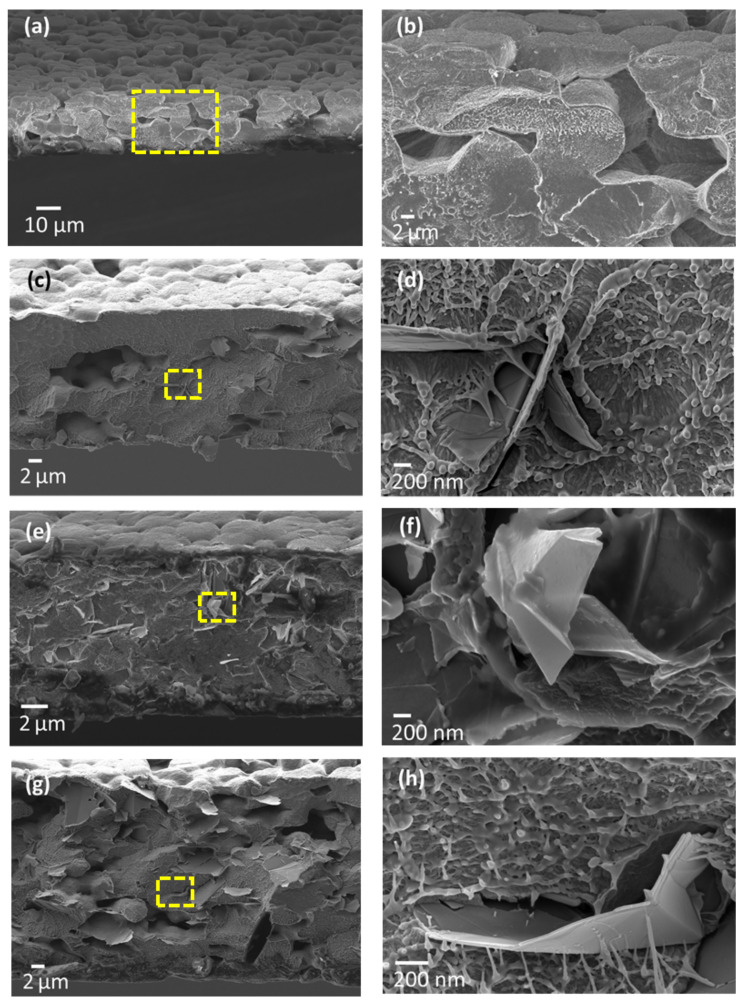
SEM images of cross sections of (**a**,**b**) neat PVDF and PVDF nanocomposite films filled with 1 (**c**,**d**), 1.5 (**e**,**f**), and 2% wt (**g**,**h**) of GNPs.

SEM micrographs showing the top surface and cross section of samples P, PG-1, PG-1.5, and PG-2 are reported in Figure 4 and Figure 5, respectively. Figure 4a,b shows that the realized neat PVDF films feature a spherulitic morphology, having high porosity and characterized by large round-shaped particles, with diameters between 10 and 15 µm, as confirmed by cross sectional images of Figure 5a,b. Upon the addition of even low concentrations of GNPs, while the spherulitic morphology is preserved, a decrease in the lateral dimensions of spherical particles is observed. Such behavior, similar to the one previously observed and discussed in [39], can be detected in Figure 4c–h, displaying the top surface’s SEM micrographs of PVDF films loaded with 1, 1.5, and 2% wt GNPs, respectively.

Moreover, when GNPs are added to the polymer, the films exhibit a marked decrease in porosity, as compared to the unfilled neat PVDF. Indeed, previous studies have shown that GNPs can trigger the nucleation of the *β* phase within the matrix, by constraining the polymer chains to orient in such a way as to promote this electroactive phase formation and to close pores [39,40]. As a consequence, as the GNP concentration is increased, PVDF composite films gradually turn from an initially semi-spherulitic structure to a more compact morphology. In particular, looking at Figure 4c,e,g and at the corresponding higher magnification micrograph (Figure 4d,f,h), it is clear that most of the original porosity has been reduced by the presence of GNPs.

Furthermore, it is worth noting that in all the produced samples a uniform dispersion of GNPs and a tight connection between the polymer matrix and GNPs are obtained. As a matter of fact, surfaces of GNPs look almost completely coated by the polymer chains (Figure 4c,e,g) and GNPs seem to be thoroughly embedded into the PVDF matrix (Figure 5d,f,h), thus suggesting an intimate contact between the nanoplatelets and the matrix.

### 3.2. Waterproof Properties

**Figure 6:**
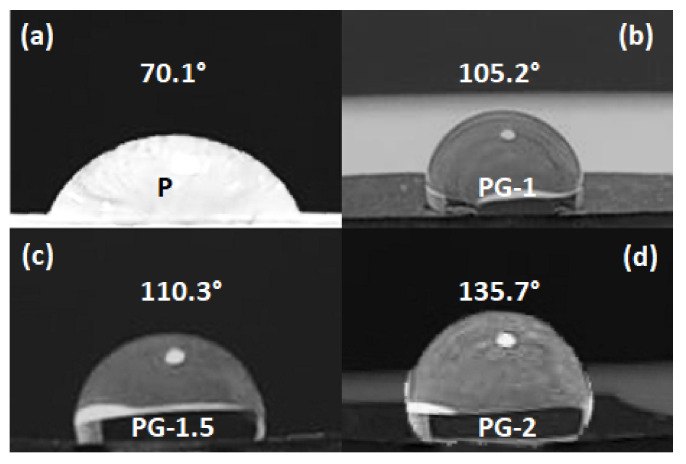
Contact angle images of sample P (**a**) and GNP-filled samples PG-1 (**b**), PG-1.5 (**c**), and PG-2 (**d**).

The wettability properties of samples P, PG-1, PG-1.5, and PG-2 are assessed through the measurement of the CA, as described in Section 2.3. As shown in Figure 6a, the CA of pure PVDF film is around 70 ± 3°.

As the GNP content increases, the CA angle rises sharply, reaching values as high as 105.2° for sample PG-1 (Figure 6b), 110.3° for sample PG-1.5 (Figure 6c), and 135.7° ± 3 for sample PG-2 (Figure 6d).

In fact, in all the considered PVDF/GNP nanocomposite films the water droplets form a semi-circular shape, showing a clear hydrophobicity. Therefore, we conclude that the selected GNP loaded nanocomposite films are hydrophobic and they are suitable for the realization of waterproof wearable sensors and their response will not be affected by moisture.

### 3.3. Mechanical Properties

**Figure 7:**
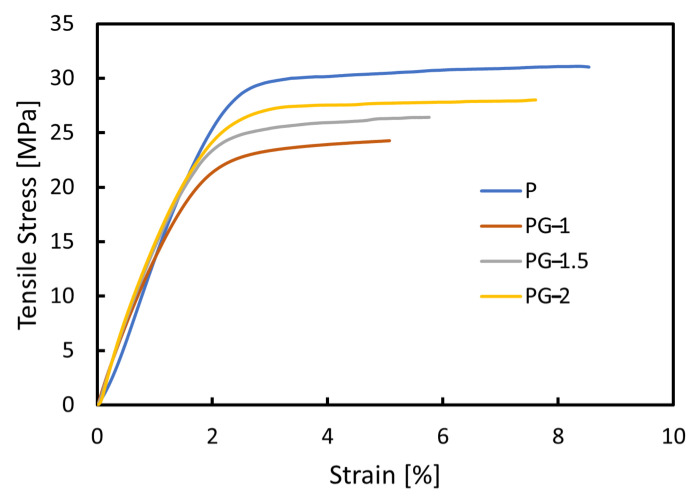
Measured stress-strain curves of samples P, PG-1, PG-1.5, and PG-2.

The measured tensile stress-strain characteristics of samples P, PG-1, PG-1.5, and PG-2 are reported in Figure 7.

The stress-strain curves, shown in Figure 7, up to the fracture points are characterized by a linear elastic region and by a plastic region, as expected since PVDF is a thermoplastic polymer.

The Young’s modulus is given by the slope of tensile stress-strain curve in the linear elastic region, i.e., before the yield zone. As the GNP concentration in the PVDF/GNP nanocomposite films increases, the Young’s modulus significantly increases. This is a further confirmation of the good adhesion and dispersion of GNPs into the PVDF matrix.

**Figure 8:**
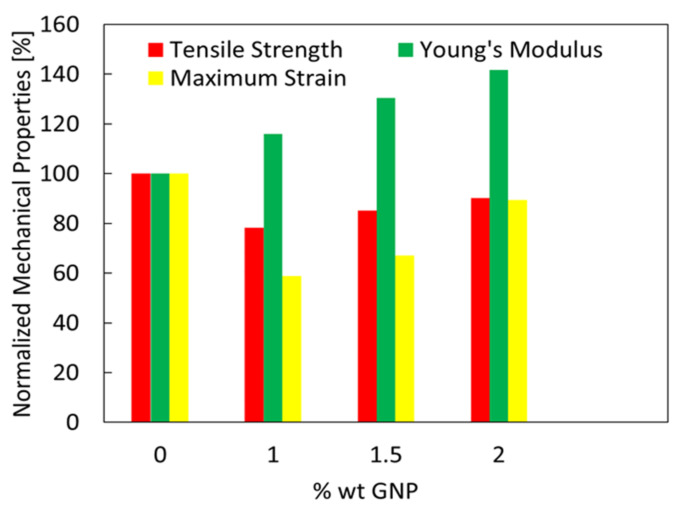
Normalized tensile strength, Young’s modulus, and maximum strain for different GNP weight fractions.

The GNP-content influence on the tensile strength, Young’s modulus, and maximum strain at break is emphasized in Figure 8, where the mechanical properties are normalized with respect to those corresponding to the neat PVDF.

Table 2 summarizes the mechanical properties of the considered samples.

### 3.4. Electrical Properties

**Figure 9:**
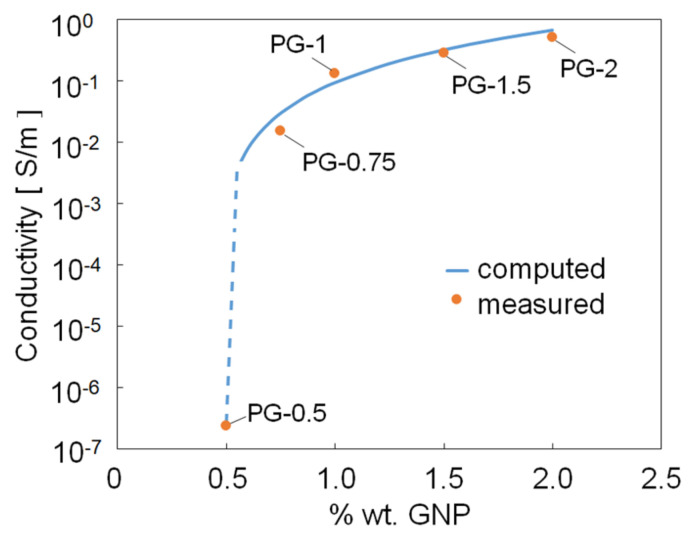
Effective dc conductivity of all the produced samples as a function of the percent GNP weight concentration.

The effective dc conductivity *γ_eff_* of all the produced PVDF/GNP samples is reported in Figure 9 with respect to the percent GNP weight concentration, ranging between 0.5 and 2% wt. As the GNP content increases, the PVDF/GNP nanocomposite films turn from the insulating to the conductive regime, due to the presence of conducting fillers embedded in an insulating polymer matrix. Electron transport is hence possible only above a filler critical concentration, known as the percolation threshold *θ*_c_. Across such filler’s concentration, the nanocomposite experiences a sudden and steep increase in the electrical conductivity due to the formation of an interconnected network of conductive GNPs [41]. Indeed, the composite effective conductivity *γ_eff_* can be modeled by means of the well-known percolation law [41,42,43].

(3) $$\gamma_{eff}=K{\left(\theta -\theta_{c}\right)}^{\tau }$$

where *θ* is the filler concentration, *τ* is the critical exponent, and *K* a determination coefficient, dependent on the material characteristics.

The best fit with the measured electrical conductivity values is obtained by setting *θ_c_* [%] = 0.55% wt, *τ* = 2.6, and *K* = 1870 S/m. Note that also samples PG-0.5 and PG-0.75 have been considered in order to evaluate the percolation threshold, even though they are not suitable for the realization of graphene-based strain sensors due to the low GNP amount.

### 3.5. Piezoresistive Characterization

**Figure 10:**
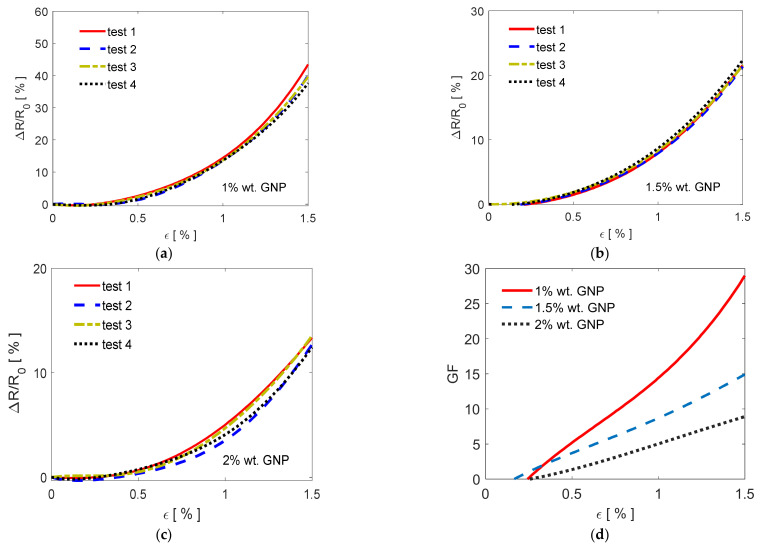
Relative resistance change Δ*R*/*R*_0_ versus the applied strain for PVDF/GNP composite films loaded at (**a**) 1, (**b**) 1.5, at (**c**) 2% wt and the corresponding (**d**) Gauge factors.

The piezoresistive behavior of samples PG-1, PG-1.5, and PG-2 is analyzed in Figure 10a–c under a quasi-static flexural loading. The graphs show the relative resistance change Δ*R*/*R*_0_ versus the applied flexural deformation *ε*. The corresponding GFs are reported in Figure 10d.

Each PVDF/GNP film sample underwent four consecutive piezoresistive tests, in order to check repeatability. The obtained data show that the piezoresistive responses are consistent and repeatable. Moreover, it is noticed that after each test the resistance assumes its initial value *R*_0_, i.e., ~294.1 kΩ for sample PG-1, ~191.8 kΩ for sample PG-1.5, and ~97.5 kΩ for sample PG-2.

Then, the resistances of the specimens increase nonlinearly as the imposed flexural deformation increases and the highest values are detected for the nanocomposite film filled with 1% wt of GNPs.

Concerning the GFs, it is observed that at flexural strains lower than ~0.2% the produced films show a dead band resulting in a negligible resistance change, as confirmed in Figure 10a–c. Then, the maximum GF at a deformation of 1.5% is nearly 30 for the sample PG-1, whereas it reaches the values of ~15 for the sample PG-1.5, and of ~8 for the sample PG-2.

Subsequently, the 1 and 2% loaded PVDF/GNP film samples undergo consecutive loading/unloading cycles for an overall duration of 20 min. The first sample is selected since it is characterized by the highest GF. On the other hand, the sample loaded at 2% wt shows a clear hydrophobicity, making it suitable for the realization of washable sensing devices.

**Figure 11:**
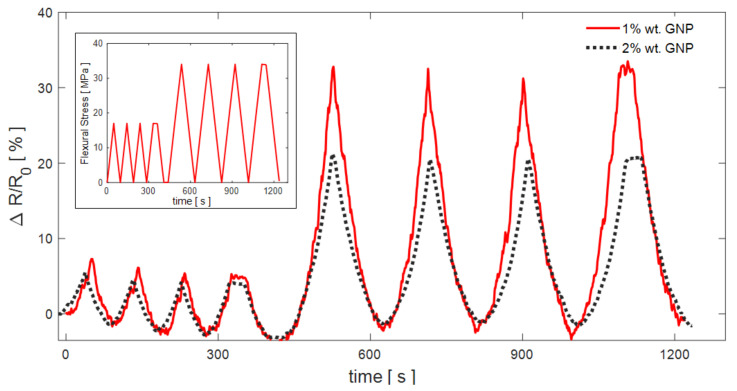
Measured variations of the relative resistance change of samples PG-1 and PG-2 due to the imposed flexural stress time-variations, shown in the figure inset.

Figure 11 reports the measured relative resistance change Δ*R*/*R*_0_ of samples PG-1 and PG-2 due to the applied flexural stress variations. The figure inset shows the imposed flexural stress with respect to time. It is worth noting that the measured relative resistance changes Δ*R*/*R*_0_ are consistent with the imposed flexural stress and that the resistance variation is more evident for the 1% loaded sample, as expected with GF being much higher.

### 3.6. Piezoresistive Graphene-Based Sensorized Glove

**Figure 12:**
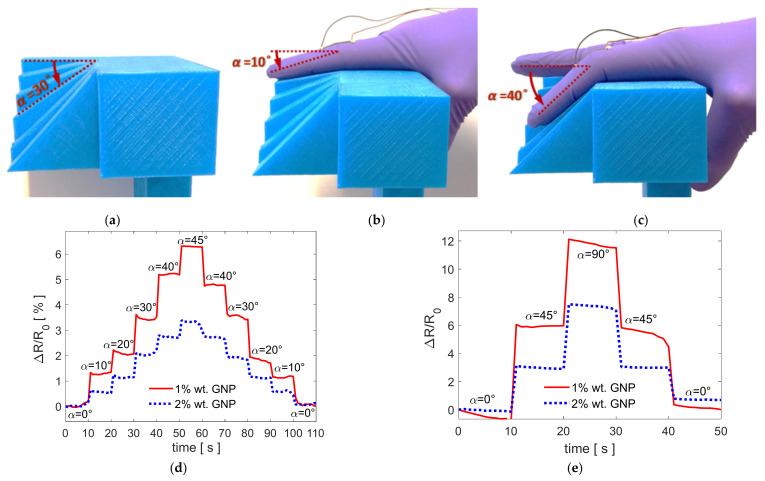
(**a**). Frame realized via 3D printing in order to rotate the proximal interphalangeal joint of an exact angle α equal to 10, 20, 30, 40, 45, and 90°. (**b**,**c**) Examples of movement detection of the index finger: The intermediate phalange is flexed with respect to the proximal one by an angle α equal to (**b**) 10 and (**c**) 40°. (**d**,**e**) Measured Δ*R*/*R*_0_ obtained by flexing the index finger every 10 s by (**d**) all the angles α between 0 and 45° and by (**e**) angles α equal to 45 and 90°.

The sensorized gloves, realized as described in Section 2.6, are tested with the aim of verifying their ability to detect proximal interphalangeal joint movements of the index finger. With this purpose, the index finger is moved by flexing the intermediate phalange with respect to the proximal one by an angle *α* between 0 and 90°. In particular, a frame, shown in Figure 12a, has been realized via 3D printing in order to rotate the proximal interphalangeal joint of an exact angle *α* equal to 10 (Figure 12b), 20, 30, 40 (Figure 12c), 45, and 90°. Simultaneously, the relative resistance change Δ*R*/*R*_0_ of the sample pasted on the glove is measured as a function of time.

Figure 12d shows the measured Δ*R*/*R*_0_ obtained by flexing the index finger every 10 s by all the considered angles *α* between 0 and 45°. Similarly, Figure 12e shows the measured Δ*R*/*R*_0_ obtained by flexing the index finger by angles *α* equal to 45 and 90°.

Looking at Figure 12d,e, it can be stated that both the realized sensorized gloves are able to detect even small proximal interphalangeal joint movements of the index finger since the measured Δ*R*/*R*_0_ profiles perfectly reproduce the index finger flexion movements. Moreover, it is noticed that the obtained data are repeatable and consistent and when the proximal interphalangeal joint is no more flexed the resistance assumes its initial value. Then, the resistance change is more evident for the sensorized glove realized with the PVDF nanocomposite film filled with 1% wt of GNPs, as expected with GF being much higher. However, it is worth noting that also the sensorized glove realized with the nanocomposite film filled with 2% wt of GNPs, characterized by higher waterproofness, is suitable for movement detection.

## 4. Discussion

**Figure 13:**
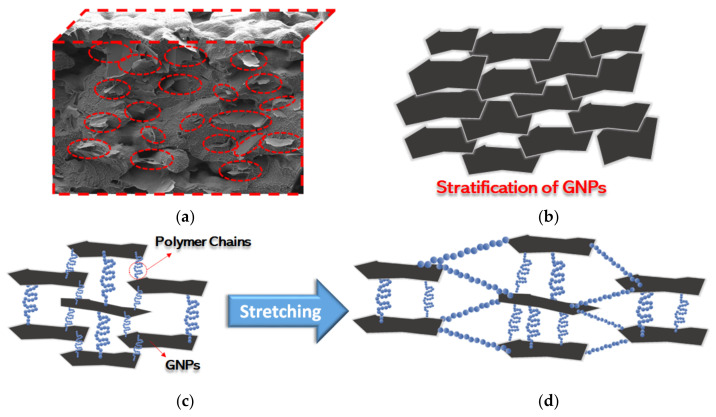
(**a**) SEM image showing GNP horizontally aligned and (**b**) corresponding schematic representation. Conducting network formed by the horizontally aligned GNPs under (**c**) normal or (**d**) stretching conditions.

The nanocomposite films are produced through an innovative production process with the scope of obtaining a perfect integration and dispersion of GNPs within the polymer matrix, without any agglomeration or defect. Such characteristics are clearly demonstrated from the performed morphological analysis, especially by the strong interaction between polymer chains and GNPs as shown in the enlargements of surface and cross-section SEM images reported in Figure 4 and Figure 5. Furthermore, it is shown that GNPs tend to stratify in the plane of the film, as highlighted in Figure 13a,b. As a consequence, the PVDF/GNP composite film morphology varies notably with respect to the one of the neat PVDF, from a round shape structure to a more compact configuration. Hence, the film porosity decreases noticeably, improving the waterproofness of the nanocomposite [28,29,30,31,32,33,34,35,36,37,38,39,40,41,42,43,44] and its mechanical and electrical characteristics [39,40]. Indeed, it has also been found that GNPs have a nucleation effect on the PVDF structure, constraining the polymer chains to orient and close pores [39,40,41,42,43,44,45].

In particular, looking at Figure 8, it is evident that the Young’s modulus significantly increases with the GNP concentration, confirming the good GNP adhesion and dispersion into the polymer matrix. In addition, the tensile strength and the percentage maximum strain of samples PG-1, PG-1.5, and PG-2 decrease slightly compared to the ones of neat PVDF films. In fact, the nanocomposite porosity decreases due to GNP loading thanks to the resulting polymer chains orientation and reorganization, leading to the semi-spherulitic morphology observed in SEM images. A slight improvement of the maximum strain of sample PG-2 with respect to sample PG-1.5 is observed, due to the fact that as the GNP amount further increases the number of pores covered by GNPs increases and mechanical properties slightly improve.

Particularly relevant is the effect of GNP addition on electrical properties. In fact, it is demonstrated that the percolation threshold $\theta_{c}$ of the obtained samples is achieved at a GNP weight fraction around 0.55% wt. This value is lower with respect to the one recently observed in case of hydrogen exfoliated graphene in a PVDF polymer matrix [26], and is comparable with the one obtained using CNTs as nanofillers [34]. Furthermore, it is worth noting that the perfect integration of GNPs inside the polymer matrix among an optimum dispersion of them, without agglomerations or defects, allowed us to reach electrical conductivity values one or two order of magnitudes higher than the ones of PVDF nanocomposites filled with other graphene-based nanofillers, such as reduced graphene oxides [13], CNTS [27], and hydrogen exfoliated graphene [26].

The obtained electrical properties allowed us to realize highly sensitive piezoresistive films suitable for the realization of strain sensors using a very low amount of GNPs. In fact, the developed specimens present a consistent and reproducible response to the applied deformation and they have outstanding sensing properties with respect to other sensors-based on the use of PVDF nanocomposite films. For instance, the nanocomposite film loaded at 1% wt of GNPs shows a relative resistance change Δ*R*/*R*_0_ around 40% at 1.5% of the imposed flexural strain, whereas the PVDF/CNT composite with the addition of poly methyl methacrylate recently presented in [27] is characterized by a Δ*R*/*R*_0_ at 5% strain of 18.6%. Furthermore, the relative resistance change recently detected using the polymer functionalized graphene-based PVDF flexible sensor is around 3.5% at 0.35% of the applied strain [26]. This is due to the stratification of GNPs, resulting in a conducting network having looser connections or fewer contacts between adjacent fillers and therefore more sensitive to an applied strain, as sketched in Figure 13c. Upon stretching (Figure 13d), the polymer chains further align themselves, enhancing the electrical resistance change, and giving rise to the exponential increase of Δ*R*/*R*_0_ with respect to the applied strain observed in Figure 10a–c.

Therefore, the produced PVDF/GNP nanocomposite films are suitable for the realization of strain sensors. Furthermore, their waterproofness and flexibility make them particularly suitable for the realization of a wearable sensing device. As a demonstration, two of the produced nanocomposite films are pasted on commercial hand gloves, realizing that wearable sensorized gloves are able to detect proximal interphalangeal joint movements of the index finger, as shown in Figure 12a,b. Specifically, a flexion movement of the intermediate phalange with respect to the proximal one by an angle α as little as 5° can be clearly detected by the realized wearable sensors. As a final remark, we note that other smart gloves equipped with carbon-based flexible nanocomposite sensors, recently presented in the literature, have not been tested for bending angles lower than 30° [5] and most of them are characterized only for fully finger bend movements [25,36]. Similarly, the response of smart gloves presented in the last years with strain sensors made of ionic liquids [38] or employing metallic nanoparticles [37] has not been characterized for angles lower than 20°.

## 5. Conclusions

In this work, new highly sensitive flexible strain sensors made of PVDF nanocomposite films loaded with GNPs are developed. The nanocomposite films are produced through an innovative production process with the scope of obtaining a perfect integration and dispersion of the GNPs inside the polymer matrix, without the formation of agglomerations or defects. Such characteristics are clearly revealed by the morphological analyses and make the nanocomposite films suitable for the realization of highly sensitive flexible strain sensors.

The produced nanocomposite films have a thickness in the 20–30 µm range, they are flexible, and are characterized by outstanding mechanical, electrical, and electromechanical properties, as well as waterproofness. Their morphology is investigated through scanning electron microscopy, the electrical and mechanical characteristics of the produced specimens are measured through standard test methods, and the piezoresistive properties are assessed through the three-point bending test, under both stationary and dynamic conditions.

The results show that the produced nanocomposite films are characterized by a consistent and reproducible response to the applied deformation. In particular, it results that the measured Gauge factor is nearly 30 and the relative resistance variation is greater than 40% at 1.5% of the applied strain for the 1% wt loaded PVDF/GNP nanocomposite film.

It is demonstrated that the developed sensors are characterized by a high and consistent response due to the optimum integration of GNPs in the matrix, especially when compared with other sensors-based on the use of PVDF nanocomposite films.

Hence, the produced specimens are cut and pasted on commercial gloves, realizing that sensorized gloves are able to detect proximal interphalangeal joint movements of the index finger. Specifically, a flexion movement of the intermediate phalange with respect to the proximal one by an angle α as little as 5° can be clearly detected by the realized wearable sensors.

As a final note, we also highlight the antibiofouling properties of PVDF, which make it particularly suitable for the considered application with respect to other types of polymers. Therefore, we can conclude that the developed sensors are particularly suitable to realize smart gloves for use in health care and for rehabilitation aid, but also for different applications, such as support in sign language translation.

## Figures and Tables

**Table 1:** GNP concentration and thickness of the produced PVDF/GNP nanocomposite films.

Sample Name	GNP Concentration [%]	Thickness [µm]
P	0	25
PG-0.5	0.5	19
PG-0.75	0.75	22
PG-1	1	36
PG-1.5	1.5	28
PG-2	2	30

**Table 2:** Mechanical properties of samples P, PG-1, PG-1.5, and PG-2.

GNP Concentration [%]	Tensile Strength [MPa]	Young’s Modulus [GPa]	Strain at Break [%]
0	31.02	1.25	8.5
1	24.27	1.45	5
1.5	26.42	1.63	5.7
2	28.01	1.77	7.6

## References

[1] Capineri L., Bulletti A. (2021). Ultrasonic Guided-Waves Sensors and Integrated Structural Health Monitoring Systems for Impact Detection and Localization: A Review. Sensors.

[2] Bidsorkhi H.C., Ballam L.R., D’Aloia A.G., Tamburrano A., De Bellis G., Sarto M.S. Flexible Graphene Based Polymeric Electrodes for Low Energy Applications. Proceedings of the 2020 IEEE 20th International Conference on Nanotechnology (IEEE-NANO).

[3] Wu X., Han Y., Zhang X., Lu C. (2016). Highly sensitive, stretchable, and wash-durable strain sensor based on ultrathin conductive layer@ polyurethane yarn for tiny motion monitoring. ACS Appl. Mater. Interfaces.

[4] Amjadi M., Kyung K.-U., Park I., Sitti M. (2016). Stretchable, skin-mountable, and wearable strain sensors and their potential applications: A review. Adv. Funct. Mater..

[5] Dong W., Yang L., Fortino G. (2020). Stretchable Human Machine Interface Based on Smart Glove Embedded With PDMS-CB Strain Sensors. IEEE Sensors J..

[6] Duan L., D’Hooge D.R., Cardon L. (2020). Recent progress on flexible and stretchable piezoresistive strain sensors: From design to application. Prog. Mater. Sci..

[7] Rahimi R., Ochoa M., Yu W., Ziaie B. (2015). Highly Stretchable and Sensitive Unidirectional Strain Sensor via Laser Carbonization. ACS Appl. Mater. Interfaces.

[8] Fortunato M., Bellagamba I., Tamburrano A., Sarto M.S. (2020). Flexible Ecoflex^®^/Graphene Nanoplatelet Foams for Highly Sensitive Low-Pressure Sensors. Sensors.

[9] Kadumudi F.B., Hasany M., Pierchala M.K., Jahanshahi M., Taebnia N., Mehrali M., Mitu C.F., Shahbazi M.-A., Zsurzsan T.-G., Knott A. (2021). The Manufacture of Unbreakable Bionics via Multifunctional and Self-Healing Silk-Graphene Hydrogels. Adv. Mater..

[10] Kumar A., Chouhan D., Alegaonkar P.S., Patro T.U. (2016). Graphene-like nanocarbon: An effective nanofiller for improving the mechanical and thermal properties of polymer at low weight fractions. Compos. Sci. Technol..

[11] Soheilmoghaddam M., Adelnia H., Bidsorkhi H.C., Sharifzadeh G., Wahit M.U., Akos N.I., Yussuf A.A. (2016). Development of Ethylene-Vinyl Acetate Composites Reinforced with Graphene Platelets. Macromol. Mater. Eng..

[12] Yang Y., Han C., Jiang B., Iocozzia J., He C., Shi D., Jiang T., Lin Z. (2016). Graphene-based materials with tailored nanostructures for energy conversion and storage. Mater. Sci. Eng. R Rep..

[13] Costa P., Nunes-Pereira J., Oliveira J., Silva J., Moreira J.A., Carabineiro S.A.C., Buijnsters J.G., Lanceros-Mendez S. (2017). High-performance graphene-based carbon nanofiller/polymer composites for piezoresistive sensor applications. Compos. Sci. Technol..

[14] Stankovich S., Piner R.D., Chen X., Wu N., Nguyen S.T., Ruoff R.S. (2006). Stable aqueous dispersions of graphitic nanoplatelets via the reduction of exfoliated graphite oxide in the presence of poly (sodium 4-styrenesulfonate). J. Mater. Chem..

[15] Hu N., Karube Y., Yan C., Masuda Z., Fukunaga H. (2008). Tunneling effect in a polymer/carbon nanotube nanocomposite strain sensor. Acta Mater..

[16] Zhu Z.H. (2015). Piezoresistive Strain Sensors Based on Carbon Nanotube Networks: Contemporary approaches related to electrical conductivity. IEEE Nanotechnol. Mag..

[17] Stankovich S., Dikin D.A., Dommett G.H., Kohlhaas K.M., Zimney E.J., Stach E.A., Piner R.D., Nguyen S.T., Ruoff R.S. (2006). Graphene-based composite materials. Nature.

[18] Sarto M.S., D’Aloia A.G., Tamburrano A., De Bellis G. (2012). Synthesis, modeling, and experimental characterization of graphite nanoplatelet-based composites for EMC applications. IEEE Trans. EMC.

[19] D’Aloia A., Marra F., Tamburrano A., De Bellis G., Sarto M. (2014). Electromagnetic absorbing properties of graphene–polymer composite shields. Carbon.

[20] Yan D.-X., Pang H., Li B., Vajtai R., Xu L., Ren P.-G., Wang J.-H., Li Z.-M. (2014). Structured Reduced Graphene Oxide/Polymer Composites for Ultra-Efficient Electromagnetic Interference Shielding. Adv. Funct. Mater..

[21] Hwang S.H., Park W.P., Park Y.B. (2013). Piezoresistive behavior and multi-directional strain sensing ability of carbon nanotube–graphene nanoplatelet hybrid sheets. Smart Mater. Struct..

[22] Wang Y., Yang R., Shi Z., Zhang L., Shi D., Wang E., Zhang G. (2011). Super-Elastic Graphene Ripples for Flexible Strain Sensors. ACS Nano.

[23] Bidsorkhi H.C., D’Aloia A.G., Tamburrano A., De Bellis G., Bracciale M.P., Santarelli M.L., Sarto M.S. Piezo-resistive properties of graphene based PVDF composite films for strain sensing. Proceedings of the 2017 IEEE 17th International Conference on Nanotechnology (IEEE-NANO).

[24] Tamburrano A., Sarasini F., De Bellis G., D’Aloia A.G., Sarto M.S. (2013). The piezoresistive effect in graphene-based polymeric composites. Nanotechnology.

[25] Li X., Koh K.H., Farhan M., Lai K.W.C. (2020). An ultraflexible polyurethane yarn-based wearable strain sensor with a polydimethylsiloxane infiltrated multilayer sheath for smart textiles. Nanoscale.

[26] Sankar V., Nambi A., Bhat V.N., Sethy D., Balasubramaniam K., Das S., Guha M., Sundara R. (2020). Waterproof Flexible Polymer-Functionalized Graphene-Based Piezoresistive Strain Sensor for Structural Health Monitoring and Wearable Devices. ACS Omega.

[27] Tang X., Pötschke P., Pionteck J., Li Y., Formanek P., Voit B. (2020). Tuning the Piezoresistive Behavior of Poly(Vinylidene Fluoride)/Carbon Nanotube Composites Using Poly(Methyl Methacrylate). ACS Appl. Mater. Interfaces.

[28] Bidsorkhi H.C., D’Aloia A.G., Tamburrano A., De Bellis G., Delfini A., Ballirano P., Sarto M.S. (2019). 3D Porous Graphene Based Aerogel for Electromagnetic Applications. Sci. Rep..

[29] Fortunato M., Bidsorkhi H.C., De Bellis G., Sarto F., Sarto M.S. Piezoelectric response of graphene-filled PVDF nanocomposites through Piezoresponse Force Microscopy (PFM). Proceedings of the 2017 IEEE 17th International Conference on Nanotechnology (IEEE-NANO).

[30] Burnham-Fay E.D., Le T., Tarbutton J.A., Ellis J.D. (2017). Strain characteristics of additive manufactured polyvinylidene fluoride (pvdf) actuators. Sens. Actuators A Phys..

[31] Gao S., Wu X., Ma H., Robertson J., Nathan A. (2017). Ultrathin multifunctional graphene-PVDF layers for multi-dimensional touch interactivity for flexible displays. ACS Appl. Mater. Interfaces.

[32] Dong W., Xiao L., Hu W., Zhu C., Huang Y., Yin Z. (2017). Wearable human–machine interface based on PVDF piezoelectric sensor. Trans. Inst. Meas. Control.

[33] Khan H., Razmjou A., Warkiani M.E., Kottapalli A.G.P., Asadnia M. (2018). Sensitive and Flexible Polymeric Strain Sensor for Accurate Human Motion Monitoring. Sensors.

[34] Vicente J., Costa P., Lanceros-Mendez S., Abete J.M., Iturrospe A. (2019). Electromechanical Properties of PVDF-Based Polymers Reinforced with Nanocarbonaceous Fillers for Pressure Sensing Applications. Materials.

[35] Zhu M., Du Z., Li H., Chen B., Jing L., Tay R.Y.J., Lin J., Tsang S.H., Teo H.T.E. (2017). Tuning electro-optic susceptibity via strain engineering in artificial PZT multilayer films for high-performance broadband modulator. Appl. Surf. Sci..

[36] Gao M., Li L., Song Y. (2017). Inkjet printing wearable electronic devices. J. Mater. Chem. C.

[37] Tian B., Yao W., Zeng P., Li X., Wang H., Liu L., Feng Y., Luo C., Wu W. (2019). All-printed, low-cost, tunable sensing range strain sensors based on Ag nanodendrite conductive inks for wearable electronics. J. Mater. Chem. C.

[38] Choi D.Y., Kim M.H., Oh Y.S., Jung S.-H., Jung J.H., Sung H.J., Lee H.W., Lee H.M. (2017). Highly Stretchable, Hysteresis-Free Ionic Liquid-Based Strain Sensor for Precise Human Motion Monitoring. ACS Appl. Mater. Interfaces.

[39] Bidsorkhi H.C., D’Aloia A.G., De Bellis G., Proietti A., Rinaldi A., Fortunato M., Ballirano P., Bracciale M.P., Santarelli M.L., Sarto M.S. (2017). Nucleation effect of unmodified graphene nanoplatelets on PVDF/GNP film composites. Mater. Today Commun..

[40] Chen J., Lu H.-Y., Yang J.-H., Wang Y., Zheng X.-T., Zhang C.-L., Yuan G.-P. (2014). Effect of organoclay on morphology and electrical conductivity of PC/PVDF/CNT blend composites. Compos. Sci. Technol..

[41] Shante V.K., Kirkpatrick S. (1985). Introduction to Percolation Theory.

[42] Boland C.S., Khan U., Ryan G., Barwich S., Charifou R., Harvey A., Backes C., Li Z., Ferreira M.S., Möbius M.E. (2016). Sensitive electromechanical sensors using viscoelastic graphene-polymer nanocomposites. Science.

[43] Ferreira A., Lanceros-Mendez S. (2016). Piezoresistive response of spray-printed carbon nanotube/poly(vinylidene fluoride) composites. Compos. Part B Eng..

[44] Bidsorkhi H.C., Adelnia H., Naderi N., Moazeni N., Mohamad Z. (2017). Ethylene vinyl acetate copolymer nanocomposites based on (un) modified sepiolite: Flame retardancy, thermal, and mechanical properties. Polym. Compos..

[45] Fortunato M., Bidsorkhi H.C., Chandraiahgari C.R., De Bellis G., Sarto F., Sarto M.S. (2018). PFM Characterization of PVDF Nanocomposite Films with Enhanced Piezoelectric Response. IEEE Trans. Nanotechnol..

